# Triplet Regimen of Metronomic Capecitabine Plus Antiangiogenic Drug and PD‐1 Inhibitor as Later‐Line Salvage Treatment for Patients With MSS/pMMR Metastatic Colorectal Cancer: A Retrospective Study

**DOI:** 10.1002/mco2.70174

**Published:** 2025-06-27

**Authors:** Qiong Yang, Yuan‐Yuan Huang, Kai‐Cong Zhang, Qiu‐Sheng Lan, Jie‐Peng Liang, Hui‐Xin Xu, Ya‐Jing Liu, Jing‐Shu Wang, Hui‐Min Liang, He‐Rui Yao, Zhong‐Hua Chu, Hai Hu

**Affiliations:** ^1^ Department of Oncology Sun Yat‐sen Memorial Hospital Sun Yat‐sen University Guangzhou China; ^2^ Guangdong Provincial Key Laboratory of Malignant Tumor Epigenetics and Gene Regulation Medical Research Center Sun Yat‐sen Memorial Hospital Sun Yat‐sen University Guangzhou China; ^3^ Department of VIP Sun Yat‐sen Cancer Center Sun Yat‐sen University Guangzhou China; ^4^ Department of Gastrointestinal Surgery Sun Yat‐sen Memorial Hospital Sun Yat‐sen University Guangzhou China; ^5^ Breast Cancer Center Zhejiang Cancer Hospital Huangzhou Institute of Medicine (HIM) Chinese Academy of Sciences Huangzhou Zhejiang China

**Keywords:** immunotherapy, metastatic colorectal cancer (mCRC), metronomic chemotherapy, microsatellite stable/mismatch repair proficient (MSS/pMMR), targeted therapy

## Abstract

Later‐line treatment has demonstrated limited survival benefits in patients with metastatic colorectal cancer (mCRC). This retrospective study evaluated the efficacy and safety of a triplet regimen combining metronomic capecitabine, antiangiogenic drugs, and PD‐1 inhibitors in patients with mCRC. Between January 2021 and December 2023, 21 patients with mCRC received a triplet regimen as later‐line treatment. Among these, seven patients achieved objective responses, nine had stable disease, two experienced disease progression, and three showed neither complete response nor progressive disease. The objective response rate (ORR) was 33.3% (7/21), and the disease control rate (DCR) was 90.5% (19/21). The median progression‐free survival (PFS) was 5.4 months (95% CI, 4.8–6.0), and the median overall survival (OS) was 10.4 months (95% CI, 6.3–14.5). A total of 17 patients experienced treatment‐related adverse events, including 9 with Grade 3/4 toxicities. After 1:1 propensity score matching, 42 patients (21 receiving the triplet regimen and 21 receiving other therapies) were included. The triplet regimen was associated with significantly improved PFS (5.4 vs. 2.7 months, *p* = 0.01) and OS (10.4 vs. 4.7 months, *p* = 0.04) compared with other therapies. In conclusion, the triplet regimen demonstrated promising antitumor activity and manageable toxicity in patients with refractory mCRC.

## Graphical abstract

A retrospective propensity score‐matched analysis demonstrated that the triplet regimen exhibited significantly improved antitumor efficacy compared to other later‐line therapies in patients with microsatellite stable/mismatch repair proficient (MSS/pMMR) metastatic colorectal cancer (mCRC). The median progression‐free survival (PFS) and overall survival (OS) were significantly prolonged in the triplet regimen group compared to the other therapies group (PFS: 5.4 vs. 2.7 months, *p* = 0.01; OS: 10.4 vs. 4.7 months, *p* = 0.04).

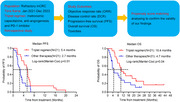

## 1 Introduction

Colorectal cancer (CRC) is among the top ten most common malignant tumors worldwide. According to the GLOBOCAN data published in 2024 [1], over 1,900,000 new cases of CRC were reported globally in 2022, ranking third in morbidity only after lung and breast cancers. Approximately 904,000 deaths were attributed to CRC, ranking it second in mortality. In China, data from the 2016 publication “2015 China Cancer Statistics” [2] indicate that CRC ranked fifth in both morbidity and mortality, with 376,000 new cases and 191,000 deaths reported. Most patients are diagnosed at the middle or advanced stages of the disease [3].

Palliative systemic therapy remains the primary treatment approach for advanced metastatic colorectal cancer (mCRC), with a median overall survival (OS) of 20–36 months reported in the literature [4, 5]. After two lines of chemotherapy, 44%–50% of patients maintain a good performance status and are suitable for further therapy to improve the quantity and quality of life [6, 7, 8, 9, 10]. However, current later‐line therapies offer limited survival benefits, highlighting the need for novel treatment strategies. The Food and Drug Administration (FDA) has approved pembrolizumab for the treatment of patients with microsatellite instability‐high (MSI‐H)/deficient mismatch repair (dMMR) mCRC [11]. Nevertheless, microsatellite‐stable (MSS) tumors account for 95% of mCRC cases, and the objective response rate (ORR) to anti‐PD‐1 monoclonal antibodies in these patients is extremely low [12, 13]. Antiangiogenic therapy has been shown to remodel the tumor immune microenvironment by increasing and activating effector immune cells, reducing inhibitory immune cells, and alleviating immune suppression, thereby potentially enhancing the efficacy of immunotherapy. The phase Ib REGONIVO trial [14] first demonstrated that adding nivolumab (a PD‐1 inhibitor) to regorafenib resulted in a higher ORR and prolonged progression‐free survival (PFS) in later‐line therapy of MSS‐type mCRC. Fruquintinib and anlotinib, small‐molecule tyrosine kinase inhibitors (TKIs) targeting VEGFR2, have also shown efficacy in mCRC. In China, fruquintinib has been approved as a late‐line treatment for mCRC [8, 9], while anlotinib has demonstrated similar efficacy to standard later‐line regimens, with an ORR of 4.26% and a PFS of 4.1 months in Phase III clinical trials [15]. Additionally, combining fruquintinib or anlotinib with PD‐1 inhibitors has shown synergistic effects in mCRC and other malignancies [16, 17]. Although the REGONIVO results were not confirmed in a series of subsequent studies conducted in the United States and China, they still demonstrated superior outcomes compared to standard later‐line regimens [18, 19, 20].

While antiangiogenic therapy has enhanced immunotherapy sensitivity in later‐line therapy of MSS‐type mCRC, efficacy remains limited. The addition of another drug on this basis is one of the current clinical research hotspots, which might remodel the tumor immune microenvironment and further enhance the efficacy of late‐line treatment for mCRC. At the 2023 ESMO congress, a Phase II study demonstrated that combining chidamide, sintilimab, and bevacizumab resulted in an ORR of 44.4% and a PFS of 7.3 months, outperforming sintilimab plus bevacizumab [21]. Another Phase II clinical trial explored the efficacy and safety of capecitabine plus antiangiogenic agents combined with PD‐1/PD‐L1 checkpoint inhibitors in salvage treatment of MSS‐type mCRC. This combination resulted in a PFS of 4.4 months (95% CI, 4.1–6.4 months), compared with 3.6 months (95% CI, 2.2–6.2 months) in the group not receiving atezolizumab (*p* = 0.07, less than the preset *p* < 0.1) [22].

This study retrospectively evaluated the efficacy and safety of a triplet regimen combining capecitabine, PD‐1 inhibitors, and antiangiogenic drugs for later‐line treatment of mCRC. The regimen achieved an ORR of 33.3% and a disease control rate (DCR) of 80.5% in the later‐line treatment of mCRC. The median PFS was 5.4 months, and the OS was 10.4 months. The most common adverse events were Grades 1 and 2 neutropenia (52.4%), followed by Grades 1 and 2 hypertension (38.1%), fatigue (33.3%), and elevated transaminases (33.3%). Grade 3 or higher hand–foot syndrome occurred in 14.3% of patients. In summary, the triplet regimen demonstrated promising tumor control rates and survival benefits in later‐line treatment of mCRC. These findings will be further validated in an ongoing Phase II study (NCT06148402), which aims to expand later‐line treatment options and improve outcomes for patients with MSS/proficient mismatch repair (pMMR) mCRC.

## 2 Results

### 2.1 Patient Characteristics

**FIGURE 1:**
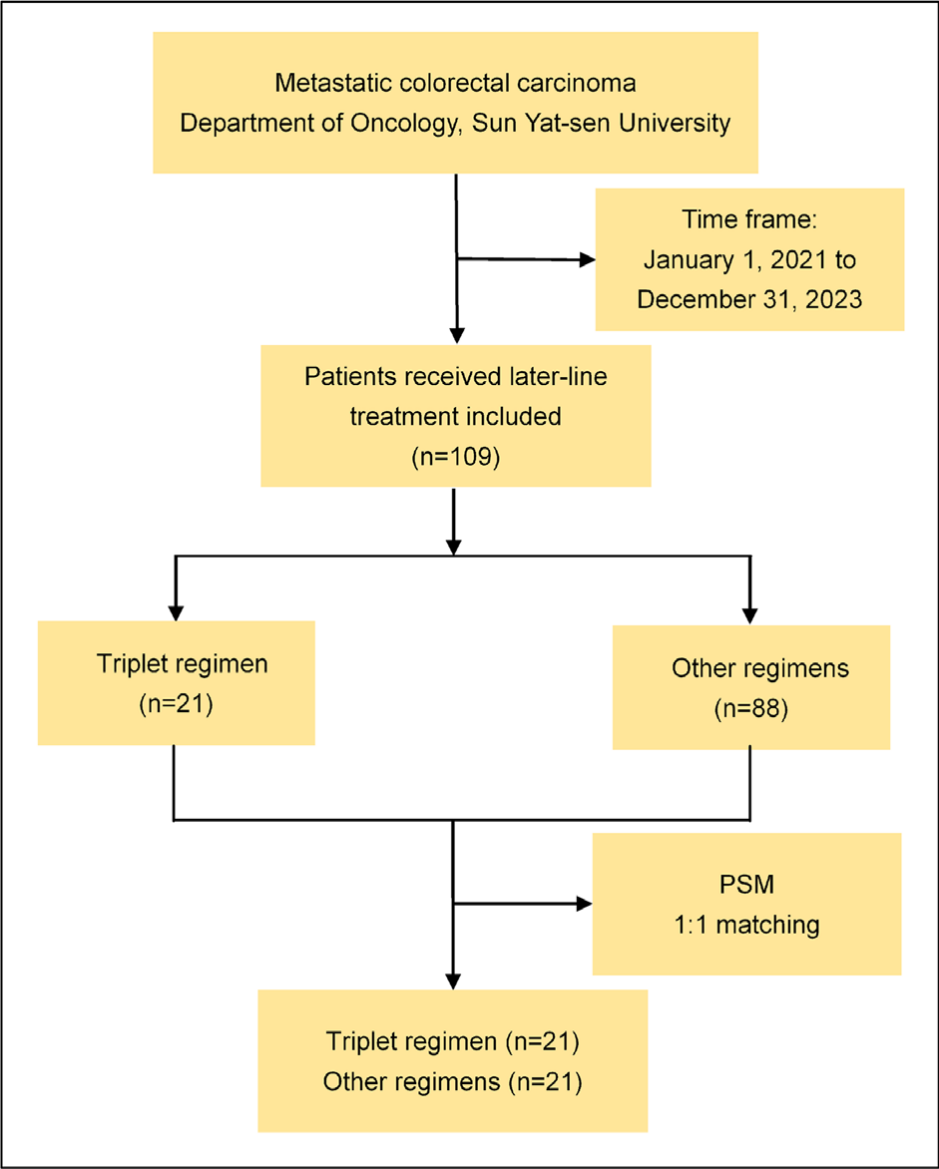
The participant flow diagram of selection and inclusion processes implemented for study subject enrollment. PSM propensity score matching.

A total of 109 patients with metastatic or advanced CRC received later‐line therapy after progression following at least two prior lines of palliative treatment at the Department of Oncology, Sun Yat‐sen Memorial Hospital, between January 1, 2021, and December 31, 2023. The patient inclusion procedure is detailed in the flow diagram shown in Figure 1. Among these, 88 patients received other therapies, including fuquinitinib, TAS‐102, regorafenib, or combinations thereof. A total of 21 patients with mCRC were treated with a triplet regimen consisting of metronomic capecitabine, small‐molecule TKIs, and PD‐1 inhibitors. Of these, 14 patients received fruquintinib, camrelizumab, and capecitabine, while 7 received anlotinib, tislelizumab, and capecitabine. All patients underwent pMMR testing by immunohistochemistry (IHC) of the primary lesion, and 18 patients were confirmed to have MSS by NGS or PCR. Metastasis biopsy samples were tested using NGS in 7 patients, and primary lesion samples were tested using NGS or PCR in 11 patients. A total of 13 patients harbored RAS gene mutations, including 3 with NRAS mutations and 10 with KRAS mutations. No BRAF or PIK3CA mutations were detected. Among the 21 patients, 15 had primary lesions on the left side and 6 on the right side. Fifteen patients had liver metastases, nine had lung metastases, and three had peritoneal or lymph node metastases. Nine patients received the experimental therapy after four prior lines of treatment, while nine received it as third‐line therapy. Three patients initiated the experimental therapy because of disease progression following first‐line FOLFOXIRI treatment. Only four patients had not previously received antiangiogenic drugs. Five patients were administered PD‐1 inhibitors before treatment initiation. Additional details regarding patient characteristics are presented in Table 1.

**TABLE 1:** Characteristics of all included patients.

Characteristics, no. (%)	Triplet regimen, *N* = 21	Other therapies, *N* = 21	*p* value
Age, years	54 (32–68)	54 (33–71)	0.93
Gender			0.75
Female	8 (38.1%)	9 (42.9%)	
Male	13 (61.9%)	12 (57.1%)	
ECOG performance status			0.82
0	7 (33.3%)	6 (28.6%)	
1	13 (61.9%)	13 (61.9%)	
2	1 (4.8%)	2 (9.5%)	
Pathological type			0.3
Adenocarcinoma	18 (85.7%)	20 (95.2%)	
Mucinous carcinoma	3 (14.3%)	1 (4.8%)	
Primary tumor site			0.73
Right	7 (33.3%)	5 (23.8%)	
Left or rectum	14 (66.7%)	16 (76.2%)	
Metastatic site			0.84
Liver	15 (71.4%)	15 (71.4%)	
Lung	9 (42.9%)	12 (57.1%)	
Liver and Lung	7 (33.3%)	10 (47.6%)	
Other	3 (14.3%)	4 (19.0%)	
RAS and BRAF status			0.75
RAS mutated	13 (61.9%)	14 (66.7%)	
BRAF mutated	0 (0%)	2 (9.5%)	
RAS/BRAF wild type	8 (38.1%)	5 (23.8%)	
Tumor mutational burden			0.64
High	1 (4.8%)	1 (4.8%)	
Low	12 (57.1%)	9 (42.9%)	
Missing data	8 (38.1%)	11 (52.3%)	
Prior lines of therapy			0.37
1 line	3 (14.3%)	0 (0%)	
2 lines	9 (42.9%)	20 (95.2%)	
≥ 3 lines	9 (42.9%)	1 (4.8%)	
Prior antiangiogenesis drugs			0.16
Yes	17 (81.0%)	16 (76.2%)	
No	4 (19.0%)	5 (23.8%)	
Prior PD‐1/PD‐L1 inhibitors			0.29
YES	5 (23.8%)	1 (4.8%)	
NO	16 (76.2%)	20 (95.2%)	
Prior chemotherapy regimens^a^			1
Oxaliplatin‐based	21 (100%)	21 (100%)	
Irinotecan‐based	21 (100%)	21 (100%)	
Fluoropyrimidine‐based	21 (100%)	21 (100%)	
Neutrocyte–lymphocyte ratio			0.82
≤ 3	12 (57.1%)	12 (57.1%)	
> 3	8 (38.1%)	7 (33.3%)	
Data missing	1 (4.8%)	2 (9.5%)	

Abbreviations: BRAF, v‐Raf murine sarcoma viral oncogene homolog B; ECOG, Eastern Cooperative Oncology Group; No., number; PD‐1, programmed cell death protein 1; PD‐L1, programmed cell death ligand 1; RAS, rat sarcoma virus oncogene.

^a^ More details are attached in Table S1.

### 2.2 Efficacy

**FIGURE 2:**
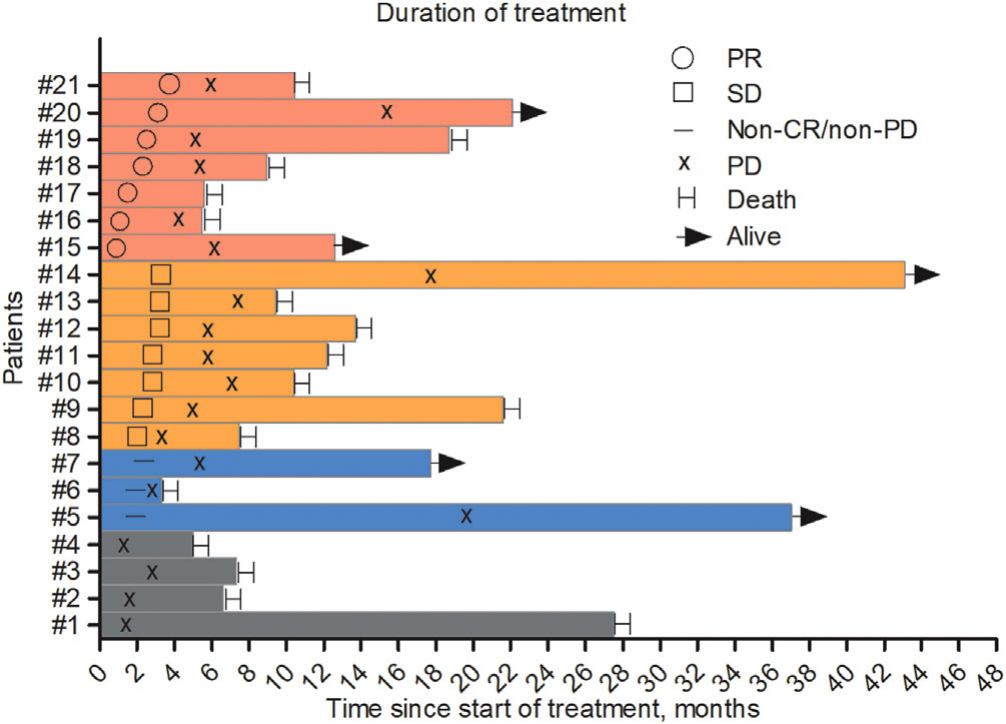
Treatment duration and corresponding radiographic responses in 21 patients who received the triplet regimen. The graphical representation employs horizontal bars to denote treatment duration, with standardized symbols indicating tumor response categories: ° partial response (PR), □ stable disease (SD), − non‐CR/non‐PD (non‐complete response/non‐progressive disease), × progressive disease (PD). Patient status at data cutoff is indicated by → (alive) and H (death).

Among the 21 patients, no complete response (CR) was observed. Seven patients achieved partial response (PR), seven had stable disease (SD), four experienced progressive disease (PD), and three had non‐CR/non‐PD. This resulted in an ORR of 33.3% and a DCR of 80.5% (Table 2, Figure 2). In the 14 patients treated with fruquintinib, camrelizumab, and capecitabine, no CR was achieved. Three patients had PR, five had SD, three had non‐CR/non‐PD, and three had PD. Among seven patients treated with anlotinib, tislelizumab, and capecitabine, no CR was achieved; four had PR, three had SD, and no PD was observed. The ORR in patients with liver metastases was 20% (3/15), significantly lower than the ORR of 66.7% (4/6) in patients without liver metastases (Table 3).

**TABLE 2:** Tumor response, TTR, and DOR in the PSM population.

Tumor response, no. (%)	Triplet regimen, *N* = 21	Other therapies, *N* = 21
CR	0 (0%)	0 (0%)
PR	7 (33.3%)	1 (4.8%)
SD	9 (42.9%)	5 (23.8%)
PD	2 (9.5%)	15 (71.4%)
Non‐CR/non‐PD	3 (14.3%)	0 (0%)
ORR, % (95% CI)	33.30%	4.80%
DCR, % (95% CI)	80.50%	28.60%
TTR (months)	2.27	2
DOR (months)	5.1	NA

Abbreviations: CR, complete response; DCR, disease control rate; DOR, duration of response; NA, not assessed; No., number; ORR, objective response rate; PD, progression disease; PR, partial response; PSM, propensity score matching; SD, stable disease; TTR, time to response.

**TABLE 3:** Progression‐free survival (PFS) and overall survival (OS) in subgroup analyses in 21 patients treated with a triplet regimen.

Characteristics	*N*	PFS (95% CI), months	*p* value	OS (95% CI), months	*p* value
Primary tumor site			0.7		0.3
Right	7	5.4 (3.8–7.0)		7.5 (3.8–11.1)	
Left or rectum	14	5.2 (3.3–7.2)		12.2 (6.2–18.1)	
Metastatic site			0.8		0.1
Liver	15	5.1 (3.6–6.6)		12.2 (6.8–17.5)	
Extrahepatic lesion	6	5.6 (5.4–5.8)		6.4 (3.1–9.6)	
Prior antiangiogenesis drugs			0.8		0.8
Yes	17	5.4 (4.7–6.1)		10.4 (4.9–15.9)	
No	4	4.0 (0.3–7.9)		9.0 (0–18.8)	
Prior PD‐1/PD‐L1 inhibitors			0.4		0.8
Yes	5	5.6 (5.6–5.7)		12.2 (6.3–18.0)	
No	16	4.2 (2.2–6.3)		9.0 (5.2–12.7)	
RAS and BRAF status			0.3		0.2
RAS mutated	13	5.6 (4.0–7.2)		12.2 (5.0–19.4)	
BRAF mutated	0			0	
RAS and BRAF wild type	8	5.1 (3.5–6.7)		9.0 (3.7–14.2)	
Neutrocyte–lymphocyte ratio (NLR)			1		0.8
≤ 3	12	5.4 (3.5–7.3)		12.2 (4.6–19.7)	
> 3	8	5.2 (4.4–6.1)		9.4 (6.1–14.7)	

Abbreviations: BRAF, v‐Raf murine sarcoma viral oncogene homolog B; PD‐1, programmed cell death protein 1; PD‐L1, programmed cell death ligand 1; RAS, rat sarcoma virus oncogene.

**FIGURE 3:**
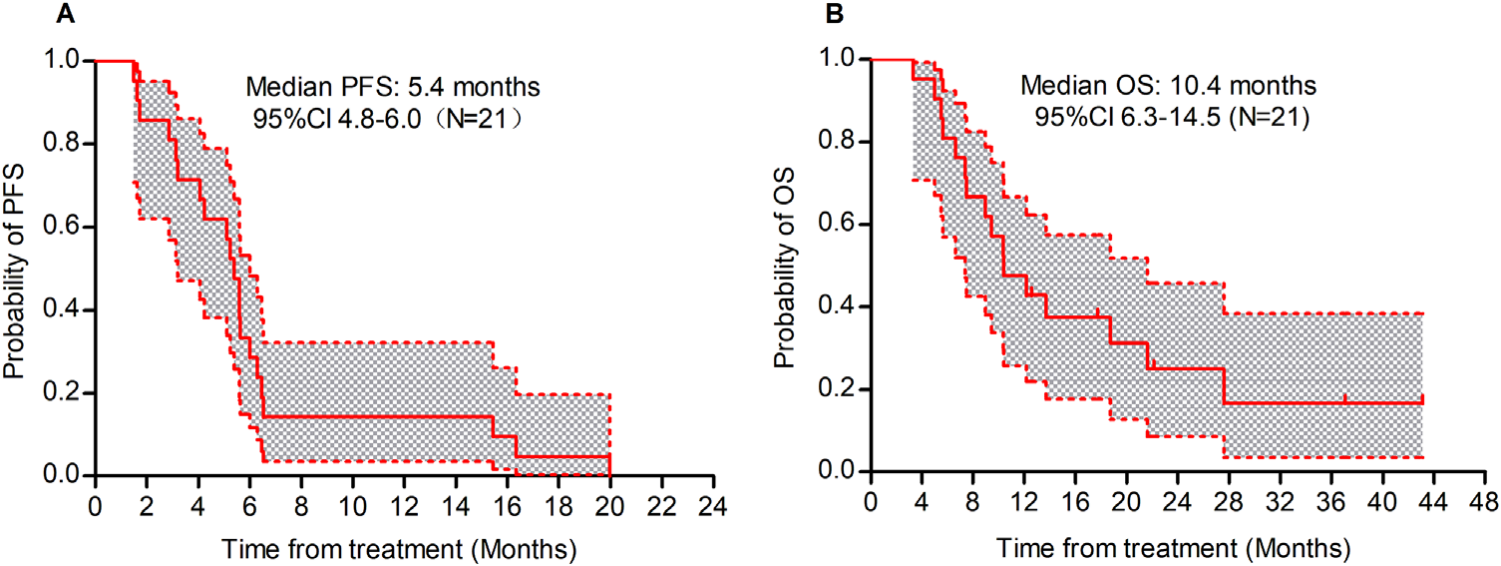
Kaplan–Meier survival analysis for patients receiving the triplet regimen. Panel A depicts the progression‐free survival (PFS) curve, while Panel B shows the corresponding overall survival (OS) curve.

Until September 30, 2024, the median PFS (Figure 3A) for all patients who received a triplet regimen was 5.4 months (95% CI, 4.8–6.0), and the OS (Figure 3B) was 10.4 months (95% CI, 6.3–14.5). Among these, the PFS for 14 patients treated with fruquintinib, camrelizumab, and capecitabine was 4.1 months, with an OS of 10.4 months. For the seven patients treated with anlotinib, tislelizumab, and capecitabine, the PFS was 5.6 months, with an OS of 9.4 months. The PFS for patients with liver metastases was 5.1 months, comparable to the 5.6 months observed in patients without liver metastases (*p* = 0.8).

**FIGURE 4:**
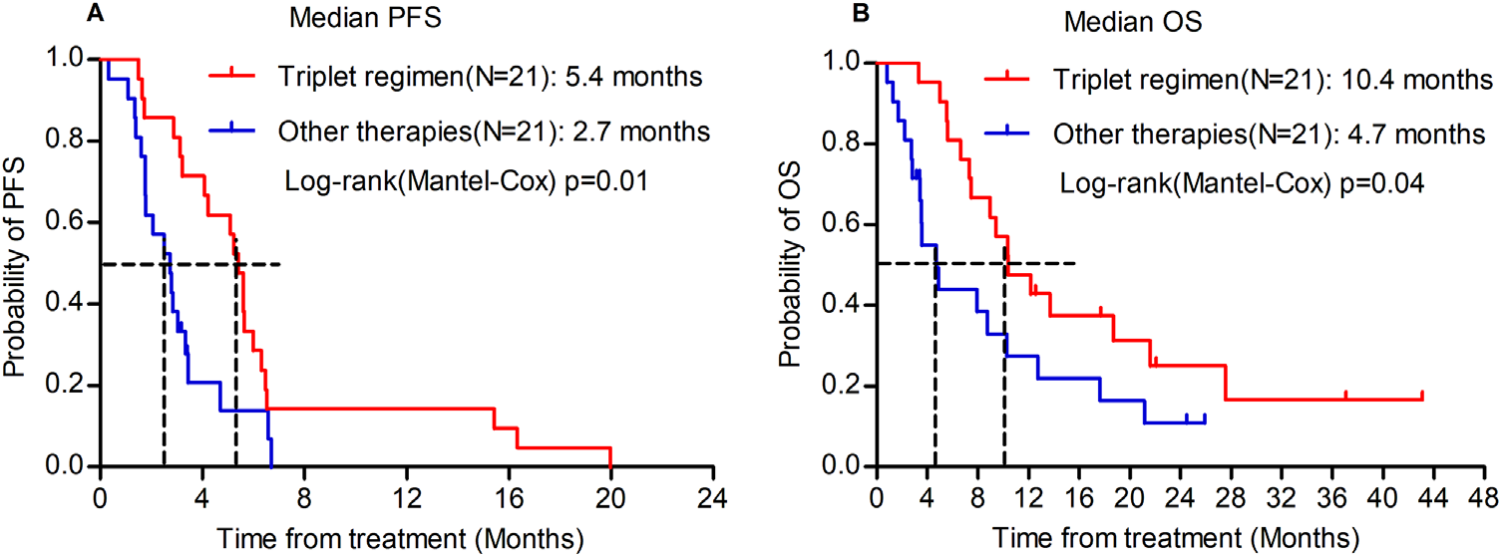
Kaplan–Meier survival curves comparing clinical outcomes between the triplet regimen and other therapy groups following propensity score matching. Panel A illustrates the comparative progression‐free survival (PFS) analysis, while Panel B presents the overall survival (OS) outcomes.

To strengthen the validity of the findings, a propensity score‐matching analysis was performed. Using a 1:1 PSM ratio, 21 patients who received other therapies were matched with 21 patients treated with the present triplet regimen (Table 1). The median PFS was 2.7 months in patients treated with other therapies, significantly shorter than the 5.4 months in patients treated with the triplet regimen (*p* = 0.01; Figure 4A). Similarly, the OS was 4.7 months in patients treated with other therapies, which was also less than 10.4 months in patients treated with the triplet regimen (*p* = 0.04; Figure 4B).

### 2.3 Subgroup Analysis

In clinical practice, factors such as primary lesion location, liver metastasis, and prior use of antiangiogenic drugs or PD‐1 inhibitors may influence patient survival outcomes. Univariate analysis revealed that Eastern Cooperative Oncology Group (ECOG) score, primary lesion location, prior bevacizumab use, prior PD‐1 inhibitors use, and liver metastasis were not significantly associated with PFS. Detailed results are presented in Table 3.

### 2.4 Toxicities

The 21 patients received 146 drug treatment cycles. No treatment‐related deaths occurred. Common treatment‐related toxicities included neutropenia (52.4%), hypertension (38.1%), fatigue (33.3%), palmar–plantar erythrodysesthesia syndrome (28.6%), aminotransferase elevation (33.3%), loss of appetite (19.0%), and hoarseness (19.0%). Grade 3 palmar–plantar erythrodysesthesia syndrome occurred in three patients (14.3%), hypertension in one patient (4.8%), and Grade 4 ulcerative colitis in one patient (4.8%). Grade 3 aminotransferase and amylase elevations were noted in one patient (4.8%). One patient discontinued the therapy because of Grade 4 immune‐related proteinuria, creatinine elevation, and ulcerative colitis (Table 4).

**TABLE 4:** Toxicities in 21 patients treated by triplet regimen.

Treatment‐related toxicities	All grades, *N* (%)	Grades 3 and 4, *N* (%)
Palmar–plantar erythrodysesthesia syndrome	6 (28.6)	3 (14.3)
Reactive cutaneous capillary endothelial proliferation	2 (9.5)	0 (0)
Fatigue	7 (33.3)	0 (0)
Loss of appetite	4 (19.0)	0 (0)
Hoarseness	4 (19.0)	0 (0)
Oral mucositis	2 (9.5)	0 (0)
Hypertension	8 (38.1)	0 (0)
Proteinuria	1 (4.8)	1 (4.8)
Creatinine elevation	2 (9.5)	1 (4.8)
Hypothyroidism	2 (9.5)	0 (0)
Hyperthyroidism	1 (4.8)	0 (0)
Ulcerative Colitis	1 (4.8)	1 (4.8)
Alanine aminotransferase elevation	2 (9.5)	1 (4.8)
Aspartate aminotransferase elevation	5 (23.8)	0 (0)
Neutrocytopenia	11 (52.4)	0 (0)
Thrombocytopenia	2 (9.5)	0 (0)
Amylase elevation	1 (4.8)	1 (4.8)

Abbreviation: N, number.

## 3 Discussion

Antiangiogenic drugs or chemotherapy alone are recommended as the standard later‐line salvage treatments for MSS/pMMR mCRC according to the NCCN/CSCO guidelines. Immune checkpoint inhibitors, as novel antitumor drugs, have demonstrated significant efficacy in various tumor types, including lung and gastric cancer [23, 24, 25, 26]. However, their efficacy as monotherapy remains limited in patients with MSS/pMMR mCRC. Both antiangiogenic drugs and chemotherapy have been shown to remodel the tumor microenvironment, potentially enhancing the efficacy of immune checkpoint inhibitors. This has become a focus of clinical exploration. Given the distinct mechanisms by which these two drug classes enhance immune responses, we hypothesized that combining both drugs with immune checkpoint inhibitors might yield superior sensitization effects. This study explored a triplet regimen of antiangiogenic drugs, PD‐1 inhibitors, and metronomic capecitabine as later‐line salvage treatment for mCRC. The results demonstrated that the triplet regimen effectively controlled tumor progression and improved PFS, irrespective of the presence of liver metastases. The ORR and DCR of this regimen in the later‐line treatment of mCRC were 33.3% and 80.5%, respectively. The median time‐to‐treatment response (TTR) was 2.27 months, PFS was 5.4 months, and OS was 10.4 months. The most common adverse events were Grades 1 and 2 neutropenia (52.4%), followed by Grades 1 and 2 hypertension (38.1%), fatigue (33.3%), and elevated transaminase levels (33.3%), all of which were Grades 1 and 2. The most frequent Grade 3 or higher adverse reaction was hand–foot syndrome, which occurred in only three cases, with an incidence of 14.3%.

A Phase II clinical trial explored a triplet regimen of capecitabine, bevacizumab, and atezolizumab as a later‐line salvage treatment for MSS‐type mCRC [22]. In this trial, patients were randomized (2:1) to receive capecitabine (850 or 1000 mg/m^2^), bevacizumab (7.5 mg/kg), atezolizumab (1200 mg; study arm), or placebo (placebo arm), with PFS as the primary endpoint. Among the 133 enrolled patients, 128 were evaluated for efficacy (82 in the study arm and 46 in the placebo arm). The median PFS in the study arm was 4.4 months (95% CI, 4.1–6.4 months) and 3.6 months (95% CI, 2.2–6.2 months) in the placebo arm (*p* = 0.07, less than the preset *p* < 0.1). The most common Grade 3 or higher treatment‐related adverse events (TRAEs) in the study arm versus the placebo arm were hypertension (six patients [7.0%] vs. two patients [4.3%]), diarrhea (six patients [7.0%] vs. two patients [4.3%]), and hand–foot syndrome (six patients [7.0%] vs. two patients [4.3%]).

Previous studies have indicated that patients with CRC and liver metastases derive less benefit from immunotherapy. Similarly, Phase II studies of bevacizumab combined with atezolizumab and capecitabine have shown limited efficacy in patients with liver metastases. The ORR was higher in patients without liver metastases (3 of 13 cases [23.1%]) than in those with liver metastases (4 of 69 cases [5.8%]), potentially due to the special immunosuppressive microenvironment of liver metastases [22]. In our study, the ORR for patients with liver metastases was 20% (3/15), significantly lower than that of the non‐liver metastasis group (66.7% [4/6]), but still higher than the ORR observed with the doublet regimen of antiangiogenic drugs combined with PD‐1 inhibitors. Interestingly, the median PFS of patients with liver metastases was 5.6 months, slightly higher than the PFS of the overall population. Two factors may explain the differences between the Phase II trial and the present study. First, the relatively small sample size in our study may have introduced bias. Second, the use of metronomic capecitabine may have contributed to the observed outcomes. Metronomic administration of capecitabine has been associated with lower toxicity, better tolerance, and modulation of the tumor microenvironment. Dr. Romiti from Italy reported that metronomic capecitabine (750 mg, BID) not only inhibits angiogenesis but also exerts immune regulatory effects, such as removing T cells (Tregs) from the tumor microenvironment. Low‐dose capecitabine may also reduce cancer stemness or selectively inhibit HIF‐1α [27]. In the non‐liver metastasis group, a higher response rate of 66.7% (4/6) and a PFS of 5.1 months were also observed with our triplet regimen. In contrast, the doublet regimen of antiangiogenic therapy combined with a PD‐1 inhibitor only showed an ORR of 10%–30% and PFS of 3.6 months in the non‐liver metastasis population. Other potential factors affecting the benefits of the three‐drug regimen, such as primary tumor location, RAS gene mutation status, history of antiangiogenic drugs and/or PD‐1 inhibitors, and history of treatment lines, were not related to efficacy.

Our study has several limitations. First, immune‐related response criteria were not used to evaluate the tumor response. These criteria are primarily designed to assess atypical immune responses that may occur during the course of immunotherapy, such as pseudoprogression and hyperprogression, which were not observed in our cohort. Therefore, RECIST criteria were suitable for evaluating objective responses in this study. Second, the OS calculation may be influenced by the fact that five patients (23.8%) remain alive. However, this is generally acceptable when the proportion of patients without survival events falls between 20% and 30%. Third, the inherent limitations of a retrospective study and the small sample size may have introduced bias. However, using 1:1 PSM, we matched 21 patients with similar clinical characteristics and found that the present triplet regimen had better PFS and OS than patients treated with other therapies.

In conclusion, our study suggests that adding capecitabine to a doublet regimen of antiangiogenic therapy and a PD‐1 inhibitor may enhance the efficacy of MSS‐type mCRC, including in patients with liver metastases, with an acceptable safety profile and no treatment‐related deaths. We are currently conducting a prospective Phase II study (NCT06148402) to further validate these findings.

## 4 Methods

### 4.1 Study Design

This retrospective study was approved by the Academic Committee of Sun Yat‐sen Memorial Hospital, Sun Yat‐sen University, and received approval from an independent ethics committee and institutional review board. Written informed consent was obtained from all participants, and no allowances or incentives were provided for participation. The study was conducted in compliance with the applicable regulations and guidelines for clinical research behavior and adhered to the ethical principles outlined in the Declaration of Helsinki.

### 4.2 Patients and Treatments

Medical records of patients who received later‐line therapy at the Department of Oncology, Sun Yat‐sen Memorial Hospital, between January 1, 2021, and December 31, 2023, were reviewed. The last follow‐up period is scheduled for September 30, 2024. Later‐line therapy was defined as treatment administered after the failure of first‐ and second‐line therapies in patients with mCRC. In this study, two types of later‐line therapies were defined: the triplet regimen and other therapies. Treatment continued until disease progression, unacceptable toxicities, patient refusal, or other reasons necessitated discontinuation. The triplet regimen consisted of TKIs (fruquintinib and anlotinib), PD‐1 inhibitors, and capecitabine. The specific regimens were as follows: fruquintinib 5 mg qd, administered continuously for 2 weeks followed by a 1‐week break; camrelizumab (PD‐1 inhibitor) 200 mg administered every 3 weeks; and capecitabine 750 mg/m^2^ bid, administered continuously for 2 weeks followed by a 1‐week break. Alternatively, anlotinib (10 mg qd) was administered continuously for 2 weeks followed by a 1‐week break; tislelizumab (PD‐1 inhibitor) 200 mg was administered every 3 weeks, and capecitabine 750 mg/m^2^ bid was administered continuously for 2 weeks followed by a 1‐week break.

The main inclusion criteria were age 18 or older, histologically confirmed CRC with metastatic disease as documented clinically or histologically, and prior treatment with fluoropyrimidine (e.g., fluorouracil or capecitabine), oxaliplatin, irinotecan with or without bevacizumab, and anti‐epidermal growth factor receptor antibody (RAS wild‐type), or intolerance/contraindications to such treatment. Prior treatment with antiangiogenic TKIs or PD‐1/PD‐L1 inhibitors did not preclude inclusion. All patients underwent pMMR testing using IHC and were confirmed to be microsatellite stable (MSS) by Next‐Generation Sequencing (NGS) and polymerase chain reactions (PCR).

### 4.3 Treatment Outcomes

The ORR was assessed by the principal investigator using the Response Evaluation Criteria in Solid Tumors (version 1.1; RECIST 1.1). Time to response (TTR) was defined as the time from treatment initiation to the first recorded PR. Duration of response (DOR) was defined as the time from treatment initiation to the first disease progression or death for any reason. PFS was defined as the time from treatment initiation to disease progression or death, whichever occurred first. OS was defined as the time from treatment initiation to death from any cause. Patients who did not experience disease progression or death at the time of data collection were considered censored cases.

TRAEs were assessed according to the National Cancer Institute Common Terminology Criteria for Adverse Events (version 5.0). Subjective toxicity information, assessed by physicians, was collected from individual patient's records. Objective toxicity information was evaluated based on examination documents and assessed by the investigator.

### 4.4 Propensity Score Matching Analysis

Propensity score matching (PSM) analysis was performed between patients treated with the triplet regimen and those treated with other therapies to enhance the validity of the findings. The propensity score model was estimated using a logistic regression model that was adjusted for variables, including primary tumor location, metastatic site, age, RAS gene mutation status, previous antiangiogenic drugs used, previous PD‐1/PD‐L1 inhibitor use, and prior lines of therapy administered. PSM was conducted using a 1:1 matching method with nearest‐neighbor matching and no caliper adjustment. A standard mean difference of less than 0.2 after matching indicated good balance.

### 4.5 Statistical Analysis

Descriptive statistics were used to summarize baseline characteristics, ORRs for tumor treatment, and toxicity data. The chi‐square test was used to compare baseline characteristics between our triplet regimen and other therapy groups. PFS and OS were estimated using the Kaplan–Meier method, with medians and 95% confidence intervals calculated. Differences in survival between groups were assessed using the log‐rank (Mantel–Cox) method. All statistical analyses were performed using R software (version 4.2.1).

## AUTHOR CONTRIBUTIONS

Q. Y., Y.‐Y. H., Z.‐H. C., and H. H. designed and supervised this project and analyzed the results; Q. Y., Y.‐Y. H., K.‐C. Z., Q.‐S. L., J.‐O. L., H.‐X. X., Y.‐J. L., J.‐S. W., and H.‐R. Y. performed treatment for patients; H.‐M. L. followed up with the patients; Q. Y. and Y.‐Y. H. wrote the paper with comments from all authors. All authors have read and approved the final manuscript.

## Ethics Statement

The human research was approved by the Medical Ethics Committee, Sun Yat‐sen Memorial Hospital (SYSKY‐2023‐773‐01, approval date: July 21, 2023). Written informed consent was obtained from all participants in this study.

## Conflicts of Interest

The authors declare no conflicts of interest.

## Supporting information



Supporting Information

## Data Availability

All data pertinent to this study, whether generated or analyzed, are comprehensively presented in this manuscript. For any additional inquiries or requests, interested parties are encouraged to contact the corresponding authors.
