# Supplementary material for: Triplet Regimen of Metronomic Capecitabine Plus Antiangiogenic Drug and PD‐1 Inhibitor as Later‐Line Salvage Treatment for Patients With MSS/pMMR Metastatic Colorectal Cancer: A Retrospective Study
Source: MedComm (2020). 2025 Jun 27;6(7):e70174. doi: 10.1002/mco2.70174 (PMC12205211; doi:10.1002/mco2.70174)
Supplement: Supplementary file 1 — Supporting Information [file MCO2-6-e70174-s001.docx]

**Triplet regimen of metronomic capecitabine plus anti-angiogenic drug and PD-1 inhibitor as later-line salvage treatment for patients with MSS/pMMR metastatic colorectal cancer: A retrospective study**

Qiong Yang^1#^, Yuan-yuan Huang^2#^, Kai-cong Zhang^1^, Qiu-sheng Lan^3^, Jie-peng Liang^1^, Hui-xin Xu^1^, Ya-jing Liu^1^, Jing-shu Wang^1^, Hui-min Liang^1^, He-rui Yao^1^, Zhong-hua Chu^3*^, Hai Hu^1,4*^

^1^Department of Oncology, Sun Yat-Sen Memorial Hospital, Sun Yat-Sen University; Guangdong Provincial Key Laboratory of Malignant Tumor Epigenetics and Gene Regulation, Medical Research Center, Sun Yat-Sen Memorial Hospital, Sun Yat-Sen University, Guangzhou 510120, China.

^2^Department of VIP, Sun Yat-Sen Cancer Center, Sun Yat-Sen University, Guangzhou 510060, China.

^3^Department of Gastrointestinal Surgery, Sun Yat-Sen Memorial Hospital, Sun Yat-Sen University; Guangdong Provincial Key Laboratory of Malignant Tumor Epigenetics and Gene Regulation, Medical Research Center, Sun Yat-Sen Memorial Hospital, Sun Yat-Sen University, Guangzhou 510120, China.

^4^Breast Cancer Center, Zhejiang Cancer Hospital, Huangzhou Institute of Medicine (HIM), Chinese Academy of Sciences, Huangzhou, Zhejiang 310022, China.

# Contributed equally

* Correspondence

**Table S1: Overview of prior therapies administered to the 21 patients preceding the triplet regimen**

| Patients | Treatment history before triplet regimen |
| --- | --- |
| #1 | First-line: 12 cycles of bevacizumab + FOLFOXIRI, BOR was SD. |
| #2 | First-line: 2 cycles of bevacizumab + XELOX, BOR was PD. Second-line: 12 cycles of cetuximab + FOLFIRI, BOR was PR. Third-line：4 cycles of mFOLFOX6，BOR was PD |
| #3 | First-line:8 cycles of bevacizumab + mFOLFOX6, BOR was SD. Second-line: 4 cycles of bevacizumab + FOLFIRI,BOR was PD |
| #4 | First-line:7 cycles of bevacizumab + mXELIRI, followed by maintenance treatment with 7 cycles of bevacizumab plus irinotecan, BOR was SD. Second-line: 4 cycles of bevacizumab + mFOLFOX6,BOR was PD |
| #5 | First-line: 4 cycles of bevacizumab + mFOLFOX6, BOR was SD. Second-line:7 cycles of bevacizumab + FOLFOXIRI, BOR was SD. |
| #6 | First-line:12 cycles of cetuximab + mFOLFOX6, followed by maintenance treatment with 6 cycles of capecitabine, the best response (BOR) was SD. Second-line: 7 cycles of bevacizumab + mXELIRI, BOR was SD. Third-line: 2 cycles of fruquintinib + camrelizumab, BOR was SD |
| #7 | First-line: 6 cycles of bevacizumab + FOLFOXIRI, BOR was SD. |
| #8 | First-line:8 cycles of XELOX, followed by maintenance treatment with 5 cycles of capecitabine, meantime liver metastatic lesion was administrated by radiofrequency ablation, BOR was SD. Second-line: 6 cycles of bevacizumab + FOLFOXIRI,BOR was SD |
| #9 | First-line: 6 cycles of XELOX, BOR was SD. Second-line: 17 cycles of cetuximab + FOLFIRI,BOR was SD. |
| #10 | First-line: 8 cycles of XELOX，BOR was SD. Second-line：6 cycles of bevacizumab + XELOX, BOR was SD. Third-line: 16 cycles of cetuximab + FOLFIRI,BOR was SD. |
| #11 | First-line: 9 cycles of bevacizumab + FOLFOXIRI, BOR was SD. Second-line: 2 cycles of apatinib + capecitabine, BOR was PD. Third-line: 16 cycles of bevacizumab + FOLFOXIRI, BOR was PR. |
| #12 | First-line: 12 cycles of bevacizumab + mFOLFOX6, BOR was SD. Second-line：19 cycles of cetuximab + FOLFIRI,BOR was SD. Third-line：4 cycles of cetuximab + irinotecan， BOR was PD. |
| #13 | First-line: 12 cycles of bevacizumab + mFOLFOX6, BOR was PR. Second-line: 8 cycles of bevacizumab + FOLFIRI,BOR was SD. Third-line: 2 cycles of TAS-102, BOR was PD. Fourth-line:2 cycles of TAS-102 + regorafenib, BOR was PD. |
| #14 | First-line: 8 cycles of bevacizumab + FOLFIRI, BOR was SD. Second-line: 2 cycles of S1 + regorafenib, BOR was PD. Third-line: 3 cycles of nivolumab + regorafenib, BOR was PD. |
| #15 | First-line: 6 cycles of XELOX, BOR was SD. Second-line: 4 cycles of bevacizumab + FOLFIRI,BOR was PD. |
| #16 | First-line: 8 cycles of bevacizumab + FOLFIRI, BOR was SD. Second-line: 2 cycles of capecitabine + regorafenib, BOR was PD. Third-line: 3 cycles of FOLFOXIRI,BOR was PD |
| #17 | First-line: 3 cycles of XELOX, followed by maintenance treatment with 5 cycles of capecitabine, BOR was PR. Second-line: 3 cycles of cetuximab + FOLFIRI, BOR was SD. Third-line: 3 cycles of raltirexed + fruquintinib, BOR was PD |
| #18 | First-line: 12 cycles of FOLFOXIRI, 4 cycles of XELOX, BOR was PR. Second-line:7 cycles of cetuximab + FOLFOXIRI, BOR was SD. |
| #19 | First-line: 5 cycles of bevacizumab + XELOX, BOR was SD. Second-line: 6 cycles of bevacizumab + FOLFIRI,BOR was SD. Third-line: 5 cycles of cetuximab + FOLFIRI,BOR was SD |
| #20 | First-line: 4 cycles of bevacizumab + FOLFOXIRI, BOR was PD. |
| #21 | First-line: 8 cycles of cetuximab + FOLFIRI, BOR was SD. Second-line:8 cycles of bevacizumab + mFOLFOX6, BOR was SD. |

mFOLFOX6: oxaliplatin, leucovorin,5-FU; BOR: best response; SD: stable disease; mXELIRI:irinotecan, capecitabine; PD: progression disease; XELOX: oxaliplatin, capecitabine; FOLFOXIRI: oxaliplatin, irinotecan, leucovirin, 5-FU; PR: partial response.
